# Supersonic shear wave imaging of the tibial nerve for diagnosis of diabetic peripheral neuropathy: A meta-analysis

**DOI:** 10.3389/fendo.2022.934749

**Published:** 2022-09-02

**Authors:** Yuping Chen, Honghong Duan, Lichun Huang, Zhengrong Jiang, Huibin Huang

**Affiliations:** ^1^ Department of Endocrinology, The Second Affiliated Hospital of Fujian Medical University, Quanzhou, China; ^2^ Department of Obstetrics and Gynecology, The Second Affiliated Hospital of Fujian Medical University, Quanzhou, China

**Keywords:** diabetic peripheral neuropathy, diagnosis, tibial nerve, stiffness measurement, supersonic shear wave imaging

## Abstract

**Background:**

Diabetic peripheral neuropathy (DPN) is the most common diabetes-associated complication and imposes a significant burden to healthcare systems. Thus, early diagnosis of DPN is extremely critical for management and outcome of diabetic patients. Supersonic Shear Wave Imaging (SSI) enables the noninvasive measurement of nerve stiffness. However, previous studies on SSI in the diagnosis of DPN were limited in sample sizes and reported various results. In this meta-analysis, we aimed to obtain comprehensive evidence on the value of tibial nerve stiffness measurement by SSI in the diagnosis of DPN.

**Methods:**

A comprehensive literature search in English and Chinese electronic database was conducted for studies (published until January 25, 2022) that investigated the diagnostic performance of tibial nerve stiffness measurement by SSI for detecting DPN. Summary receiver operating characteristics (SROC) modelling was constructed to conduct the meta-analysis of diagnostic accuracy of SSI for detecting DPN.

**Results:**

Finally, a total of 12 eligible studies with 1325 subjects were included for evaluation, and a meta-analysis was conducted to evaluate the diagnostic performance of tibial nerve stiffness measurement by SSI for detecting DPN. For tibial nerve stiffness measurement by SSI, the summary sensitivity and specificity for the diagnosis of DPN were 80% (95% confidence interval [CI]: 73%–86%) and 86% (95% CI: 82%–89%), respectively. The summary area under the ROC curve (AUROC) value of the SROC was 0.90 (95% CI: 0.87–0.92), for diagnosing DPN. A subgroup analysis of 11 SSI studies from China revealed similar diagnostic performance, with a summary sensitivity of 79% (95% CI: 72%–85%), specificity of 86% (95% CI: 82%–89%) and summary AUROC value of the SROC of 0.90 (95% CI: 0.87–0.92) for diagnosing DPN.

**Conclusions:**

Our meta-analysis suggests that a tibial nerve stiffness measurement by SSI shows good performance in diagnosing DPN and has considerable potential as a noninvasive tool for detecting DPN.

## Introduction

Diabetes mellitus (DM) is a chronic progressive metabolic disease, which is characterized by systemic hyperglycemia (1). The incidence and prevalence of both type 1 and type 2 DM is increasing rapidly throughout the world and becoming a major public health problem (2, 3). Over the past 20 years, the number of adults living with diabetes has more than tripled (4). By 2045, the number of people with diabetes worldwide is expected to rise to 700 million (5). According to International Diabetes Federation (IDF) Atlas (9th edition), in 2019 alone, approximately 4.2 million deaths worldwide were attributable to diabetes and its complications (5).

The final outcome of diabetes is a variety of chronic complications (1). Among these, diabetic peripheral neuropathy (DPN) is the most common diabetes-associated complication (6). Notably, there is a high incidence of DPN in the diabetic population. The previous study has reported that approximately 50% of people with diabetes will develop DPN (7). If it is not timely diagnosed and treated, it may cause further serious complications, such as foot ulcers, gangrene, and amputation (8, 9). Importantly, this condition continues to have a great impact on the quality of life of people with diabetes and also imposes a significant burden to healthcare systems (10, 11). In fact, DPN is known as the strongest initiating risk factor for diabetic foot ulceration (12, 13). Thus, early diagnosis of DPN is extremely critical for management and outcome of diabetic patients.

Currently, there are several methods, including quantitative sensory testing (e.g., monofilament examination), clinical neurological function scoring system (e.g., Toronto clinical scoring system), high-frequency ultrasound and nerve conduction studies (NCS), are commonly used to detect DPN (10, 14). Among these, NCS is considered to be one of the gold standard methods for the diagnosis of DPN (15). However, this technique also has disadvantages; for instance, it is expensive, time-consuming, and requires professional doctors to operate, making it difficult to implement in many primary care settings (10, 14). As a consequence, there is a strong need for a simple and effective tool for detecting DPN.

Recently, measurement of nerve stiffness by ultrasound elastography becomes a noninvasive alternative method for the diagnosis of DPN. Ultrasound elastography is a promising imaging technology, which measures the speed of a shear wave, providing a quantitative estimate of tissue stiffness (16). Three types of ultrasound elastography, including transient elastography, point shear wave elastography and two-dimensional shear wave elastography, have now been commercialized (17). In contrast, two-dimensional shear wave elastography represents a novel quantitative ultrasound elastography technique, which uses an acoustic radiation force impulse to generate shear waves (18). This technique has several strengths; for example, it enables a simultaneous display of a color-coded elastographic map on a conventional B-mode ultrasound image in time, and to visualize the stiffness of the examined areas (19, 20). Notably, among different ultrasound equipments installed with this technique, the estimates of the speed of a shear wave in the same nerve are widely heterogeneous. Supersonic Shear Wave Imaging (SSI; Aix-en-Provence, France) is one of the most widely used equipments of two-dimensional shear wave elastography for the evaluation of neuromuscular pathologies, including DPN, to the best of our knowledge.

Some studies have reported on the value of SSI for diagnosing DPN. However, previous studies on SSI in the diagnosis of DPN were limited in sample sizes and reported various results. Therefore, in this meta-analysis, we aimed to obtain comprehensive evidence on the value of tibial nerve stiffness measurement by SSI in the diagnosis of DPN. Additionally, we also conducted a subgroup analysis to summarize the diagnostic performance of SSI studies from China. In the present study, only relevant data from the SSI was collected for evaluation and meta-analysis.

## Methods

Preferred Reporting Items for Systematic Reviews and Meta-Analyses (PRISMA) guidelines for performing and reporting our current meta-analysis were followed (21).

### Search strategy

A computerized search was conducted using the four English electronic databases (PubMed, EMBASE, Web of Science, and Cochrane Library) and four Chinese electronic databases (China National Knowledge Infrastructure, Chinese Biomedical Database, Chinese VIP Information Database, and Wanfang Med Database) to identify studies (published until January 25, 2022) that investigated the diagnostic performance of tibial nerve stiffness measurement by SSI for detecting DPN. The following search terms were used: ((diabetes) OR (diabetic peripheral neuropathy) OR (diabetic complications)) AND ((shear wave elastography) OR (supersonic shear wave imaging)). Moreover, references of the identified articles were also examined for other relevant publications. We used EndNote X9 software (Clarivate Analytics, Philadelphia, PA, United States) to import and manage retrieved records.

### Inclusion and exclusion criteria

Overall, the diagnostic performance of studies that investigated the accuracy of tibial nerve stiffness measurement by SSI for detecting DPN was considered eligible for our meta-analysis. Specifically, studies were included in the present meta-analysis according to the following criteria: (1) the study examined the accuracy of the tibial nerve stiffness measurement by SSI for diagnosing DPN; (2) the study enrolled more than 30 patients; and (3) the study provided sufficient data and therefore allowed us to construct at least one 2 × 2 table for test performance. The exclusion criteria were as follows: (1) studies were not relevant to the topic; (2) studies were performed on patients without DM; (3) studies had incomplete data sets; (4) duplicate publications (we excluded the studies with the smaller population); and (5) non-original research articles, including guidelines, conference abstracts, patents, reviews, protocols and commentary. Of note, only studies that used the SSI were included in this analysis, we therefore excluded other types of ultrasound elastography techniques, such as transient elastography and point shear wave elastography, and other ultrasound equipments for two-dimensional shear wave elastography technique, such as Siemens Virtual Touch tissue imaging and quantification (VTIQ) and Toshiba shear wave elastography.

### Data extraction and quality assessment

In this meta-analysis, we extracted the following data from the included studies: (1) study characteristics, including author, year of publication, region, number of study population, etc.; (2) patients’ data, including age, patient gender, body mass index (BMI), etc.; (3) SSI characteristics, including probe, number of measurements for each subject, number of readers, representative value of elasticity (median or mean), blinding to the reference standard, and interval time between the reference standard and SSI; and (4) the reference standard used for diagnosing DPN.

The revised Quality Assessment of Diagnostic Accuracy Studies-2 (QUADAS-2) tool (22) was used to assess the quality of the studies included in this meta-analysis. Two reviewers (Y.P. Chen and H.H. Duan) of our research independently evaluated the study eligibility and quality and extracted the outcome data, with a third reviewer (H.B. Huang) adjudicating on disagreements.

### Data synthesis and analysis

Statistical analysis was performed by Stata version 15 (STATA Corp., College Station, TX, USA) and Meta-Disc software version 1.4. In this meta-analysis, our main purpose was to investigate the performance of tibial nerve stiffness measurement by SSI for the diagnosis of DPN. The data was extracted from the included studies and the 2 × 2 table for test performance was constructed, which was then used to calculate the sensitivity, specificity, positive and negative likelihood ratio and diagnostic odds ratio (DOR). Moreover, the summary receiver operating characteristic (SROC) curve was also constructed. We then examined the summary area under the ROC curve (AUROC) and the corresponding 95% confidence intervals (CIs) to further assess the accuracy of tibial nerve stiffness measurement by SSI for the diagnosis of DPN. Furthermore, a subgroup analysis was also conducted to evaluate the diagnostic performance of SSI studies from China.

Cochran’s Q-test (*P* < 0.1 indicated significant heterogeneity) and inconsistency index (I^2^) was used to assess the non-threshold heterogeneity of the results between SSI studies in this meta-analysis (23). The inconsistency index I^2^ was calculated in our analysis and then used to qualify the amount of non-threshold heterogeneity. It may be considered to represent moderate, substantial or considerable heterogeneity if the inconsistency index I^2^ value ≥ 30%, ≥ 50% or ≥ 75%. In addition, the Spearman correlation coefficient was also calculated in order to evaluate the threshold effect of the included studies. A *P* value < 0.05 was considered to indicate the existence of a threshold effect. Publication bias was assessed using the Deeks’ funnel plots (24). When *P* value < 0.05, the results were suggested that significant publication bias existed.

## Results

### Literature search

**Figure 1:**
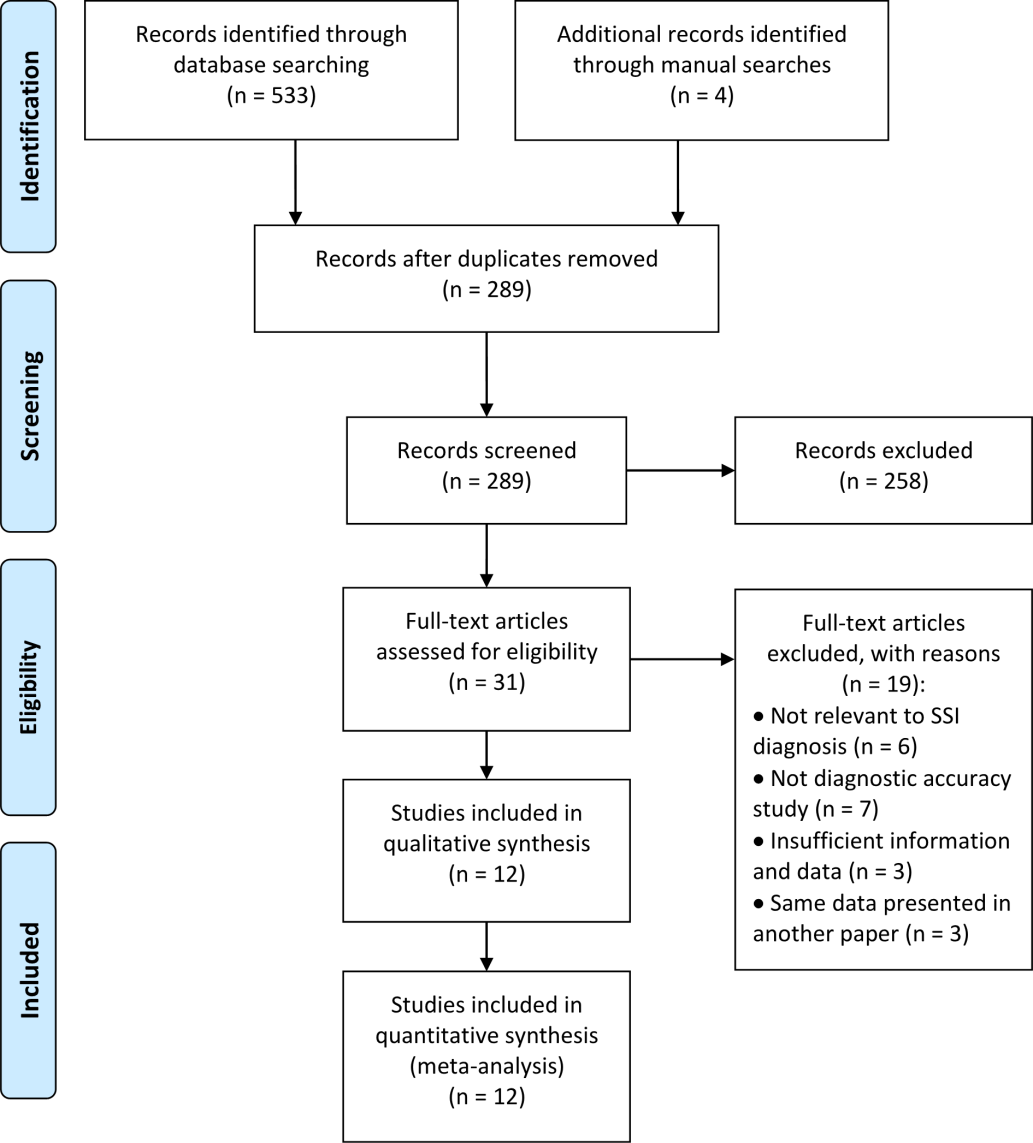
Flowchart of study selection.

**Figure 1** shows a flow diagram for the literature selection process. Using the above-mentioned search strategy, a total of 537 records were retrieved. After removal of 248 duplicates, 289 records were retained. However, 277 studies were excluded for some reasons, such as conference abstracts, patents, reviews, protocols, studies were not relevant to the topic, etc. Finally, 12 eligible studies (25–36) were ultimately reserved for evaluation and meta-analysis. Specifically, five of these studies were retrieved from the English database and 7 from Chinese database.

### Study and technical characteristics

**Table 1** demonstrates the basic characteristics of the studies included in this meta-analysis. Among the included studies, 10 (83.3%) studies were published between 2019 and 2021. With the exception of one study from the Turkey, the vast majority of published studies were from China. In total, 1325 subjects were included in this meta-analysis for evaluation. Specifically, they were 472 DPN patients, 25 clinically defined DPN patients (i.e., patients with clinical signs or symptoms of DPN but normal NCS), 408 non-diabetic peripheral neuropathy (NDPN) patients, and 420 control participants. In the DPN and NDPN groups, the study populations were confirmation of type 2 DM patients.

**Table 1:** Basic characteristics of the included studies.

Author, year, region	Duration of patient recruitment	Group	Size	Median/mean age, years	Male/female	BMI, kg/m^2^
Dikici, 2017, Turkey	Nov 2013–Jul 2014	DPN	20	60.0	10/10	31.4
		NDPN	20	61.0	8/12	29.8
		Control	20	58.0	9/11	28.7
Huang, 2018, China	Dec 2016–Oct 2017	DPN	62	NA	34/28	NA
		NDPN	50	NA	24/26	NA
		Control	52	NA	26/26	NA
Jiang, 2019, China	Nov 2017–May 2018	DPN	25	66.2	11/14	24.3
		CDDPN	25	60.9	6/19	24.2
		NDPN	20	57.1	8/12	25.4
		Control	20	57.8	10/10	24.2
He, 2019, China	Nov 2016–Jul 2017	DPN	40	60.4	17/23	25.1
		NDPN	40	58.6	22/18	24.7
		Control	40	55.2	24/16	22.4
Zhao, 2019, China	Feb 2017–Feb 2018	DPN	30	58.4	15/15	24.1
		NDPN	30	57.8	15/15	24.7
		Control	30	59.3	15/15	23.4
Wang, 2019, China	Sep 2017–Feb 2018	DPN	61	56.3	34/27	NA
		NDPN	58	55.8	29/29	NA
		Control	60	55.6	28/32	NA
Chen, 2020, China	Oct 2018–Aug 2019	DPN	30	54.4	18/12	25.7
		NDPN	33	54.9	15/18	26.2
		Control	33	51.5	14/19	23.3
Tang, 2020, China	Jan 2019–Dec 2019	DPN	50	56.2	27/23	24.3
		NDPN	50	55.1	24/26	25.0
		Control	50	57.5	29/21	24.8
Su, 2020, China	May 2018–Aug 2019	DPN	30	53.6	16/14	NA
		NDPN	30	51.5	17/13	NA
		Control	30	52.3	15/15	NA
Huang, 2020, China	Jan 2016–Dec 2016	DPN	39	53.5	NA	NA
		NDPN	35	52.7	NA	NA
		Control	32	51.9	NA	NA
Wang, 2021, China	Dec 2017–Dec 2019	DPN	41	59.1	28/13	24.7
		NDPN	42	58.5	27/15	24.8
		Control	21	56.1	8/13	23.5
Li, 2021, China	Jan 2020–Jun 2020	DPN	44	55.4	31/13	24.7
		Control	32	51.7	16/16	23.7

BMI, body mass index; CDDPN, clinically defined diabetic peripheral neuropathy; DPN, diabetic peripheral neuropathy; NA, not available; NDPN, non-diabetic peripheral neuropathy.

The technical characteristics of the SSI technique used in the included studies are summarized in **Table 2**. Most of the included studies were used a 4–15 MHz linear array transducer (10, 83.3%), when performing examinations with SSI technique. In addition to one study, 11 studies used the mean value to determine the representative value of elasticity. As the measure of tibial nerve stiffness by SSI, 10 studies used elasticity (kilopascals), and two studies used the shear wave speed (meters per second).

**Table 2:** Characteristics of SSI technique in the included studies.

Author, year, region	Probe	No. of measurements	Representative values	Readers	Blinding	Time interval	Reference
Dikici, 2017, Turkey	4–15 MHz linear-array transducer	3	Mean	2	Yes	1 week	NCS
Huang, 2018, China	4–15 MHz linear-array transducer	3	Mean	NA	NA	< 2 days	Electrophysiology tests
Jiang, 2019, China	4–15 MHz linear-array transducer	4	Mean	2	Yes	NA	NCS
He, 2019, China	4–15 MHz linear-array transducer	3	Mean	2	Yes	NA	NCS
Zhao, 2019, China	4–15 MHz linear-array transducer	6	Mean	1	NA	Same day	Electrophysiology tests
Wang, 2019, China	4–15 MHz linear-array transducer	3	Mean	1	Yes	NA	Electrophysiology tests
Chen, 2020, China	4–15 MHz linear-array transducer	5	Mean	NA	NA	NA	Electrophysiology tests
Tang, 2020, China	4–15 MHz linear-array transducer	3	Mean	1	NA	Same day	Electrophysiology tests
Su, 2020, China	NA	NA	NA	NA	NA	NA	Electrophysiology tests
Huang, 2020, China	4–15 MHz linear-array transducer	3	Mean	NA	NA	NA	NA
Wang, 2021, China	4–15 MHz linear-array transducer	3	Mean	1	Yes	1 week	Electrophysiology tests
Li, 2021, China	9-14 MHz linear-array transducer	NA	Mean	1	Yes	NA	NA

NA, not available; NCS, nerve conduction study; SSI, supersonic shear wave imaging.

### Diagnostic performance of SSI for the detection of DPN

In total, 12 studies (with 472 DPN patients, 25 clinically defined DPN patients, 408 NDPN patients, and 420 control participants) were included for the quantitative analysis to investigate the accuracy of tibial nerve stiffness measurement by SSI for the diagnosis of DPN.

**Figure 2:**
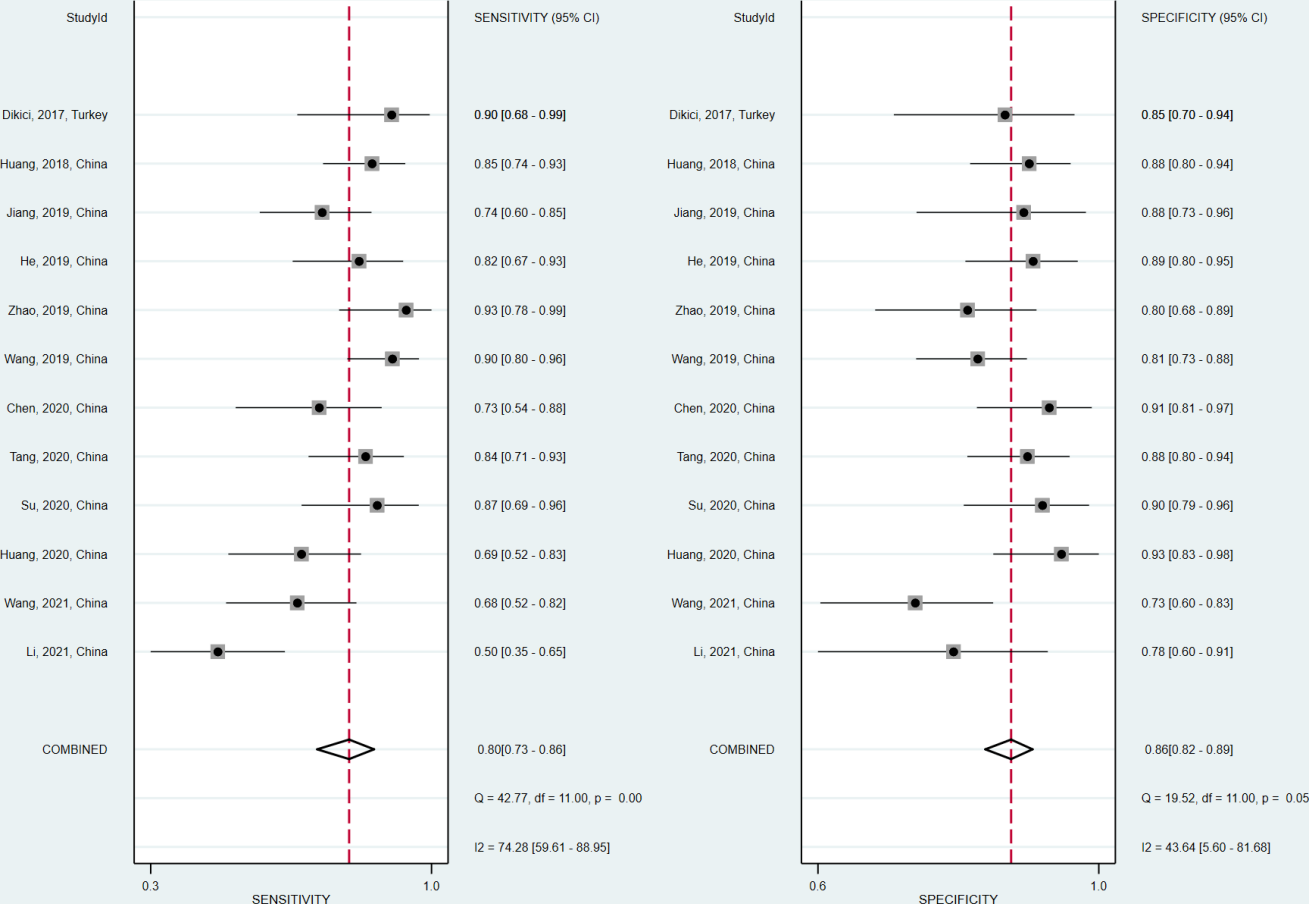
Coupled forest plots of the sensitivity and specificity of tibial nerve stiffness measurement by Supersonic Shear Wave Imaging (SSI) for diagnosing diabetic peripheral neuropathy (DPN).

**Figure 3:**
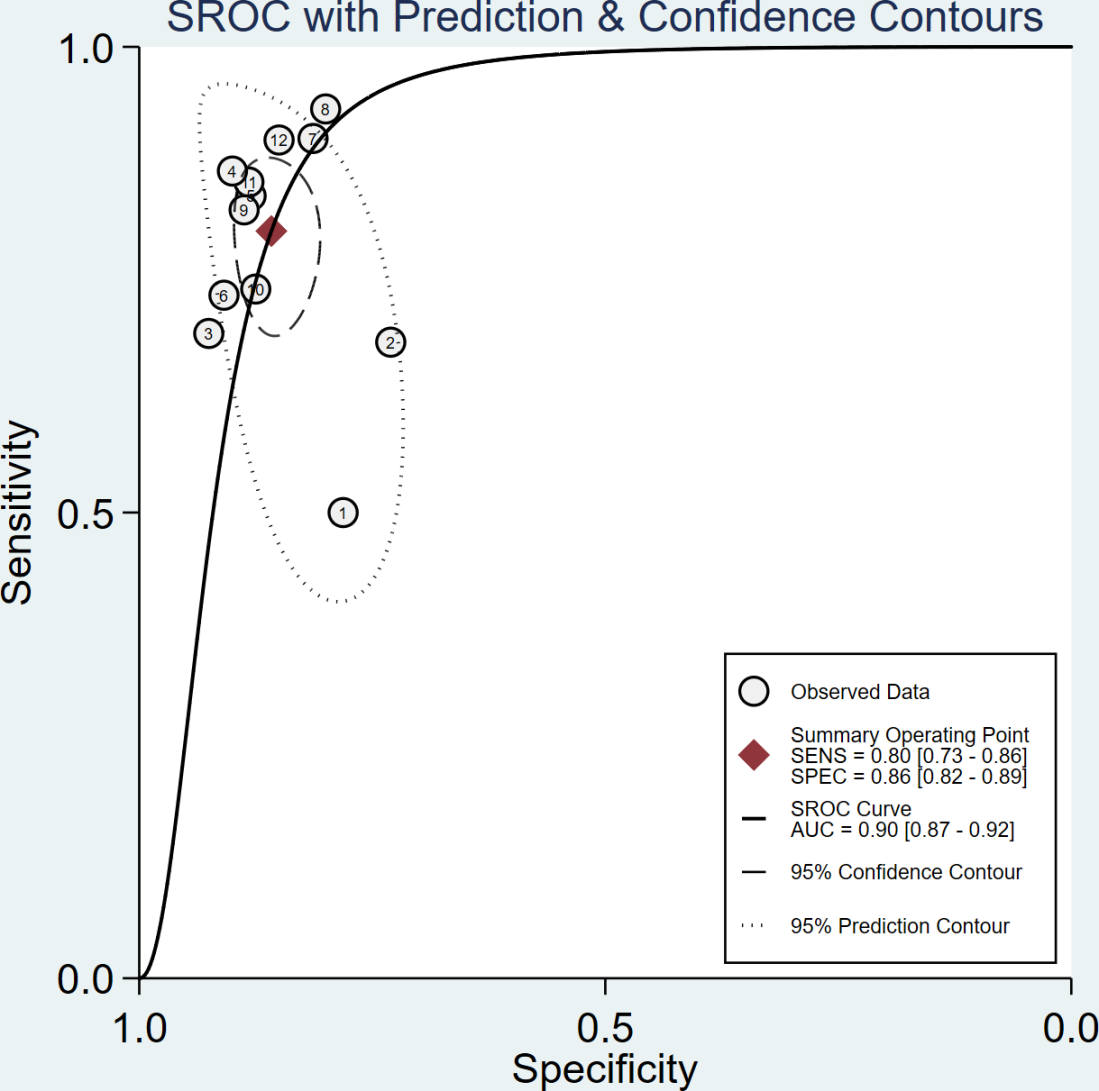
Summary receiver operating characteristic (SROC) curve of tibial nerve stiffness measurement by Supersonic Shear Wave Imaging (SSI) for diagnosing diabetic peripheral neuropathy (DPN).

The sensitivity and specificity of the included studies ranged from 50.0% to 94.2% and 73.8% to 92.9%, respectively, as shown in **Table 3**. **Figure 2** demonstrates that, for tibial nerve stiffness measurement by SSI, the summary sensitivity and specificity for the diagnosis of DPN were 80% (95% CI: 73%–86%) and 86% (95% CI: 82%–89%), respectively. The mean AUROC value of tibial nerve stiffness measurement by SSI for diagnosing DPN was 0.880 (range, 0.634–0.949). When the results were combined, the summary AUROC value of the SROC was 0.90 (95% CI: 0.87–0.92), as shown in **Figure 3**. Moreover, we found that the summary DOR of tibial nerve stiffness measurement by SSI was 25 (95% CI: 15–41; **Table 4**), for diagnosing DPN.

**Table 3:** Summary of diagnostic performance of SSI for diagnosing DPN.

Author, year, region	Optimal EI outcome	Cut-off value	AUROC	Sensitivity, %	Specificity, %
Dikici, 2017, Turkey	Mean	51.1 kPa	0.941	90.0	85.0
Huang, 2018, China	Mean	50.1 kPa	0.936	85.0	88.0
Jiang, 2019, China	Min	45.7 kPa	0.867	74.0	87.6
He, 2019, China	Mean	4.1 m/s	0.927	81.3	88.7
Zhao, 2019, China	Mean	47.4 kPa	0.946	94.2	80.0
Wang, 2019, China	Mean	52.5 kPa	0.928	90.0	81.7
Chen, 2020, China	Mean	32.7 kPa	0.902	73.3	90.9
Tang, 2020, China	Mean	46.7 kPa	0.940	84.0	88.0
Su, 2020, China	NA	59.3 kPa	0.949	86.7	90.0
Huang, 2020, China	Max	60.6 kPa	0.878	69.2	92.9
Wang, 2021, China	Mean	71.3 kPa	0.712	68.3	73.8
Li, 2021, China	Max	3.4 m/s	0.634	50.0	78.1

AUROC, area under the receiver operating characteristic curve; DPN, diabetic peripheral neuropathy; EI, elasticity indices; NA, not available; SSI, supersonic shear wave imaging.

**Table 4:** Meta-analysis results of tibial nerve stiffness measurement by SSI for prediction of DPN.

	Number of studies (Subjects)	Summary sensitivity (95% CI, %)	Summary specificity (95% CI, %)	Summary LR+ (95% CI)	Summary LR- (95% CI)	Summary AUROC (95% CI)	Summary DOR (95% CI)
SSI							
Tibial nerve	12 (1325)	80 (73–86)	86 (82–89)	5.7 (4.4–7.2)	0.23 (0.16–0.32)	0.90 (0.87–0.92)	25 (15–41)

AUROC, area under the receiver operating characteristic curve; CI, confidence interval; DOR, diagnostic odds ratio; DPN, diabetic peripheral neuropathy; LR+, positive likelihood ratio; LR-, negative likelihood ratio; SSI, supersonic shear wave imaging.

A subgroup analysis of 11 SSI studies from China revealed similar diagnostic performance. The results showed that the summary sensitivity and specificity of tibial nerve stiffness measurement by SSI for diagnosing DPN were 79% (95% CI: 72%–85%) and 86% (95% CI: 82%–89%). The summary AUROC value of the SROC was 0.90 (95% CI: 0.87–0.92), for diagnosing DPN. Detailed results are provided in **Supplementary Materials**.

### Heterogeneity and publication bias

**Figure 4:**
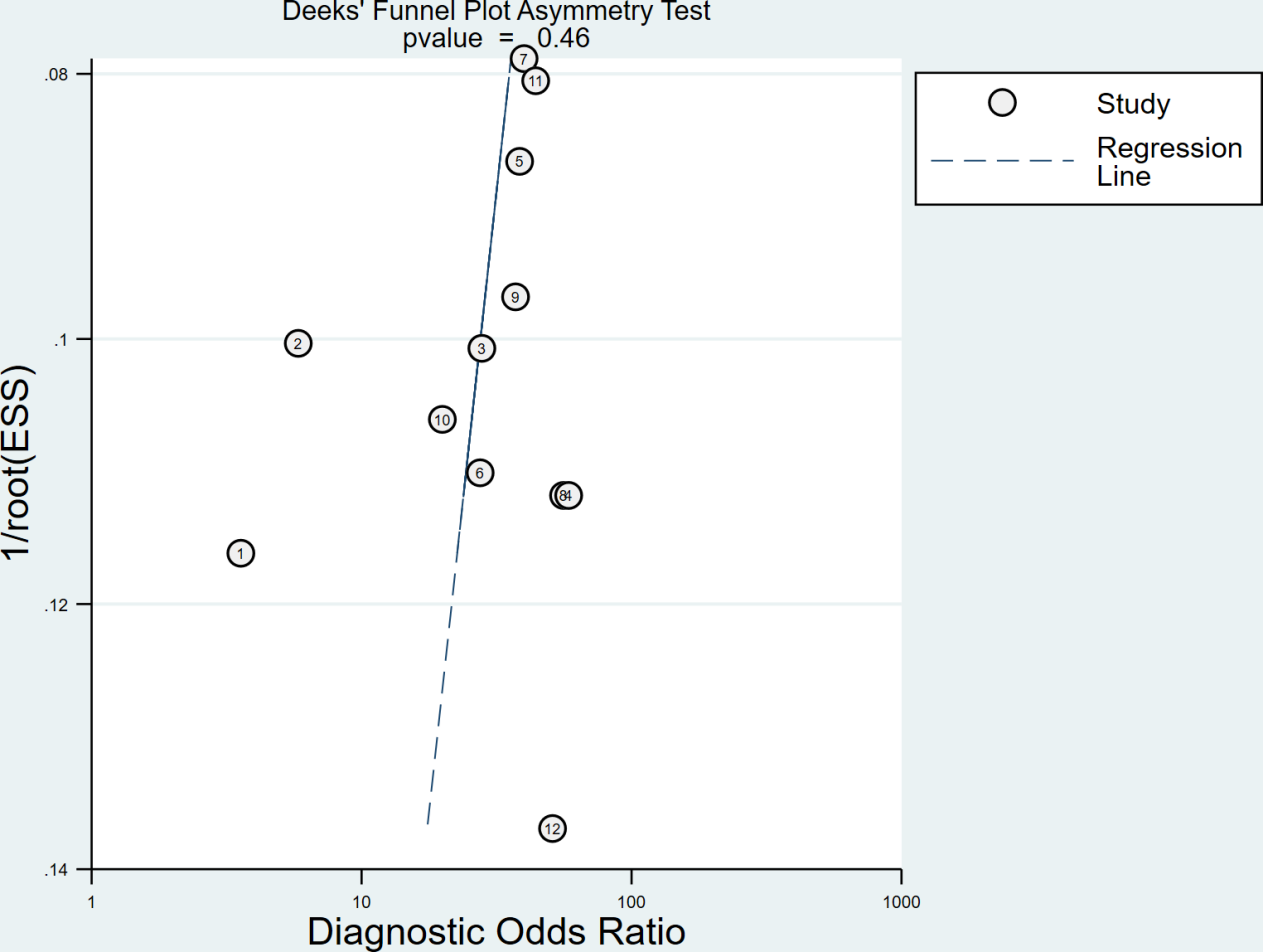
Deeks’ funnel plot used to assess publication bias.

Spearman correlation coefficient was calculated to evaluate the threshold effect of the included studies. We found that there was no threshold effect among the SSI studies, as depicted by the Spearman correlation coefficient of 0.042 (*P* = 0.897). Cochran’s Q-test and I^2^ statistic showed substantial heterogeneity with regard to the summary sensitivity (I^2^ = 74.28%, *P* < 0.1) and moderate heterogeneity with regard to the summary specificity (I^2^ = 43.63%, *P* < 0.1). There was no evidence of publication bias in SSI studies in diagnosing DPN in this meta-analysis (*P* = 0.46), as shown in **Figure 4**.

## Discussion

In the present study, we only collected relevant data from the SSI for evaluation and meta-analysis and excluded other types of ultrasound elastography, and therefore, this major source of heterogeneity may be avoided. Based on a systematically literature search in English and Chinese electronic database, we aimed to obtain comprehensive evidence on the value of tibial nerve stiffness measurement by SSI in the diagnosis of DPN.

In this meta-analysis, we identified 12 eligible studies to assess the performance of tibial nerve stiffness measurement by SSI for diagnosing DPN. Pooled analysis of 12 included studies showed that a tibial nerve stiffness measurement by SSI exhibited good diagnostic performance, with a summary sensitivity of 80% (95% CI: 73%–86%), specificity of 86% (95% CI: 82%–89%), and AUROC of 0.90 (95% CI: 0.87–0.92). A subgroup analysis of 11 SSI studies from China revealed similar diagnostic performance, it showed that the summary sensitivity and specificity were 79% (95% CI: 72%–85%) and 86% (95% CI: 82%–89%), respectively, and the summary AUROC was 0.90 (95% CI: 0.87–0.92). Our findings thus indicate that SSI, as a novel ultrasound elastography technique, can be used as a noninvasive tool for diagnosing DPN.

In recent years, ultrasound elastography has gained traction as a new imaging technique for obtaining tissue biomechanical characteristics (18, 37). SSI is a novel as well as one of the most widely used ultrasound elastography for the evaluation of neuromuscular pathologies, which allows real-time visualization of tissue viscoelastic properties beyond conventional B mode ultrasound, and shear wave velocity or Young’s modulus for region of interest can be obtained quantitatively and dynamically (38). SSI has some potential advantages over other types of ultrasound elastography techniques, such as transient elastography and point shear wave elastography (37, 38). Indeed, previous studies have revealed that in diabetic patients, the tibial nerve is stiffer when measured with SSI (25, 35). Even in diabetic patients without DPN, the tibial nerve stiffness was significantly higher than in control subjects (25, 35). This suggests that SSI-based stiffness measurements of the nerve can reflect the nerve changes in diabetic patients indirectly.

Electrophysiological examinations are still the cornerstone of neuropathy diagnosis, which can be provide detailed information about the dysfunction of affected nerves (39). Notably, this technique mainly evaluates the changes of the large nerve fibers, while in DPN patients, small nerve fibers are the first to be affected (10, 40). Compared to the controls, the tibial nerve is stiffer in NDPN groups when measured with SSI (25, 35). It may appear earlier than can be seen in abnormal electrophysiological examinations. Therefore, SSI has potential value as an effective tool in the early detection of subelectrophysiological DPN (35).

It is necessary to mentioned that heterogeneity was found across the studies included for the present meta-analysis. This may be due to several factors, such as characteristics of study population, methods of DPN diagnosis and SSI measurements, and sometimes a variable distribution of the severity of diabetes. In the study conducted by Wang et al. (35), authors concluded that no significant correlation between the tibial nerve stiffness and the DM duration was existed, regardless of the DPN history. However, other factors, such as age and height of patients (27, 41), may have potential influence on the measurement of tibial nerve stiffness by SSI. Moreover, ultrasound machine transducer and its orientation, and other technical considerations and artifacts may also influence the resulting values when performing SSI measurements (18). We did not conducted meta-regression and subgroup analyses to explore these confounding factors, however, by the small number of studies included in this meta-analysis. As a consequence, more research is needed in the future.

Some limitations of this meta-analysis are noteworthy. First, in addition to the tibial nerve, the most frequently involved site (25), DPN may affect many more peripheral nerves, such as the median nerve and sural nerve. DPN is a multiple peripheral nerve disease; therefore, future studies will be needed for the investigation of other nerves and multiple anatomic regions of the same nerve using SSI (28). Second, all but one of the studies included in this meta-analysis were based on Chinese patients, and the results may not be generalizable to other populations. A prospective, multicenter investigation with a large sample is thus warrented. Third, in this meta-analysis, we did not studied the relationship of the stiffness of the tibial nerve and the degree of diabetic neuropathy. Finally, the optimal cut-off value of the tibial nerve on SSI for diagnosing DPN was not established. In order to ensure its applicability to clinical use, further work is required to identify the optimal cut-off value for SSI.

In conclusion, our meta-analysis has suggested that a tibial nerve stiffness measurement by SSI shows good performance in diagnosing DPN, which provides more information for clinical treatment. Therefore, SSI has considerable potential as a noninvasive tool for detecting DPN. Notably, the cut-off values for detecting DPN with this method still vary greatly across the included studies. Further work is required to identify the optimal cut-off value for SSI of the tibial nerve to ensure its applicability to clinical use.

## Data availability statement

The raw data supporting the conclusions of this article will be made available by the authors, without undue reservation.

## Author contributions

YC contributed to the study design, YC and HD contributed to the literature search. YC, LH and ZJ completed the data analysis. YC and HH generated the figures and tables. YC completed the manuscript. YC and HH proofread the manuscript. All authors contributed to the article and approved the submitted version.

## Funding

This work was supported by Sciences and Technology project of Fujian Provincial Department (2019J01166), Innovative medical research project of Fujian Province (2018-CX-33) and The Second Affiliated Hospital of Fujian Medical University horizontal scientific research project (HX202201 and HX202202).

## Conflict of interest

The authors declare that the research was conducted in the absence of any commercial or financial relationships that could be construed as a potential conflict of interest.

## Publisher’s note

All claims expressed in this article are solely those of the authors and do not necessarily represent those of their affiliated organizations, or those of the publisher, the editors and the reviewers. Any product that may be evaluated in this article, or claim that may be made by its manufacturer, is not guaranteed or endorsed by the publisher.
