# Supplementary material for: Supersonic shear wave imaging of the tibial nerve for diagnosis of diabetic peripheral neuropathy: A meta-analysis
Source: Front Endocrinol (Lausanne). 2022 Sep 2;13:934749. doi: 10.3389/fendo.2022.934749 (PMC9478111; doi:10.3389/fendo.2022.934749)
Supplement: Supplementary file 1 [file DataSheet_1.pdf]

Table S1. Meta-analysis results of diagnostic performance of SSI studies from China.

	Number of Studies (Subjects)	Summary Sensitivity (95% CI, %)	Summary Specificity (95% CI, %)	Summary LR+ (95% CI)	Summary LR- (95% CI)	Summary AUROC (95% CI)	Summary DOR (95% CI)
SSI							
Tibial nerve	11 (1265)	79 (72–85)	86 (82–89)	5.6 (4.3–7.3)	0.24 (0.17–0.34)	0.90 (0.87–0.92)	23 (14–40)

Abbreviations: AUROC, area under the receiver operating characteristic curve; CI, confidence interval; DOR, diagnostic odds ratio; LR, likelihood ratio; SSI, supersonic shear wave imaging.

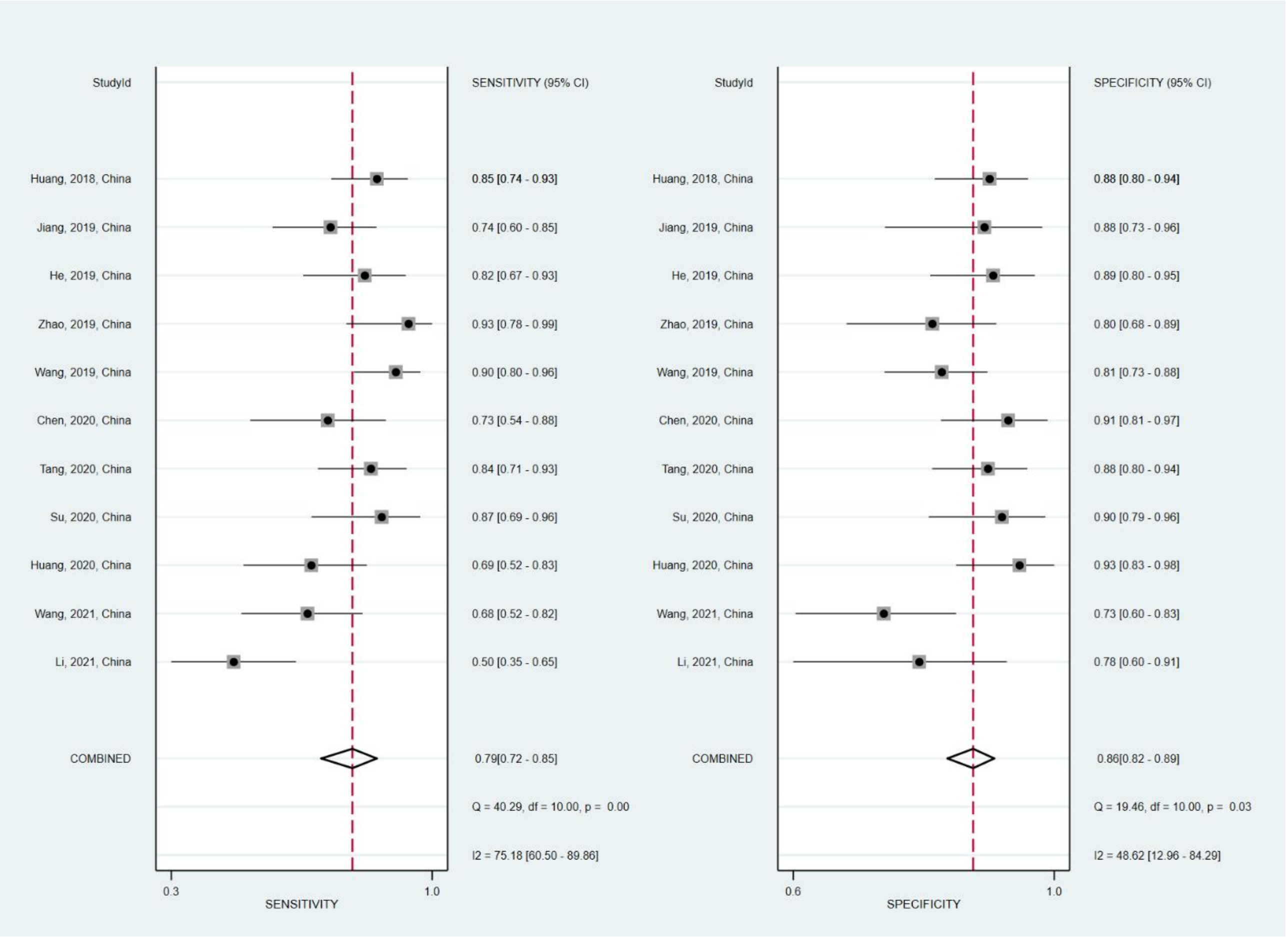

Figure S1. Coupled forest plots of the sensitivity and specificity of diagnostic performance of Supersonic Shear Wave Imaging (SSI) studies from China.

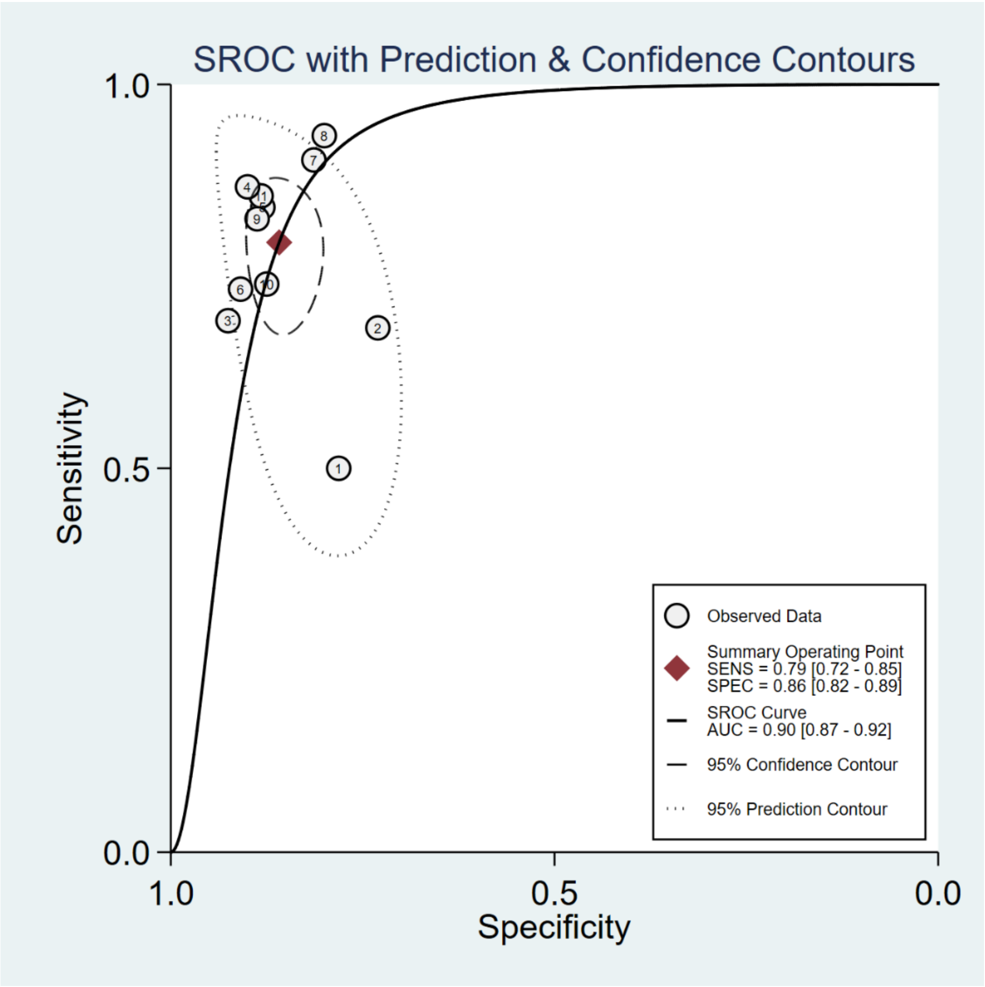

Figure S2. Summary receiver operating characteristic (SROC) curve of diagnostic performance of Supersonic Shear Wave Imaging (SSI) studies from China.
